# Right Atrial Thrombus After Cardiac Surgery With Tissue Expander in a Child With Right Pulmonary Agenesis

**DOI:** 10.1016/j.atssr.2025.10.005

**Published:** 2025-11-11

**Authors:** Hirofumi Haida, Yukihiro Yoshimura, Akihiro Shimotakahara

**Affiliations:** 1 Department of Cardiovascular Surgery, Keio University School of Medicine, Tokyo, Japan; 2 Department of Cardiovascular Surgery, Tokyo Metropolitan Children’s Medical Center, Tokyo, Japan; 3 Department of Surgery, Tokyo Metropolitan Children’s Medical Center, Tokyo, Japan

## Abstract

We report the case of a child with right pulmonary agenesis, congenital tracheal stenosis, and atrial septal defect (ASD). At age 2 years, an intrathoracic tissue expander was placed to reduce tracheal compression from mediastinal shift and to enable subsequent atrial septal defect closure. At age 5 years, a right atrial mass was incidentally detected along with right atrial compression caused by the expander. Emergency mass resection was performed, and the mass was diagnosed as a thrombus. The tissue expander was subsequently deflated and removed to prevent further compression. The patient had no recurrence of thrombus or respiratory symptoms during 8-year follow-up.

The Video can be viewed in the online version of this article [https://doi.org/10.1016/j.atssr.2025.10.005] on https://www.annalsthoracicsurgeryshortrep.org

Pulmonary agenesis is a rare congenital anomaly frequently accompanied by malformations of the cardiovascular and respiratory systems.^1^ The resulting mediastinal shift can cause tracheal compression and distorted intrathoracic anatomy, complicating surgical interventions.^2^ Intrathoracic tissue expanders have been used in selected cases to reposition the mediastinum and relieve airway obstruction.^3^ While they may offer short-term benefits, such as improved airway patency and enhanced surgical exposure, their long-term hemodynamic effects and potential complications remain inadequately understood.

We report a case of a child with right pulmonary agenesis, tracheal stenosis, and atrial septal defect (ASD). This case demonstrates the dual benefit and risk of using an intrathoracic tissue expander in a child with right pulmonary agenesis requiring cardiac surgery. The expander facilitated both airway decompression and surgical exposure, but contributed to delayed right atrial thrombus formation due to chronic compression. This case highlights both the utility and potential complications of mediastinal repositioning devices, raising important considerations for their long-term use and relevance in the era of minimally invasive pediatric cardiac surgery.

A male infant was born at term with a prenatal diagnosis of right pulmonary agenesis, confirmed postnatally along with dextrocardia, mediastinal shift, congenital tracheal stenosis, and ASD. He had mild respiratory symptoms, including exertional wheezing, and was managed conservatively with medication. Growth and neurodevelopment were age-appropriate.

At 2 years old, cardiac catheterization revealed a pronounced left-to-right shunt (Qp/Qs 2.1), elevated right ventricular end-diastolic volume (213% of normal), and pulmonary artery pressure of 50/9 mm Hg. Given the risk of developing pulmonary hypertension, surgical ASD closure was indicated. Catheter-based closure was not feasible due to an inadequate aortic rim (2.5 mm). Preoperative computed tomography revealed moderate congenital tracheal stenosis (50% narrowing, 60% of tracheal length) and compression of the trachea by the stretched ascending aorta and transverse arch due to mediastinal shift. To relieve tracheal compression and ensure airway patency during and after surgery, placement of a tissue expander was planned to reposition the mediastinum.

Under general anesthesia, a right posterolateral thoracotomy was performed, and a INTEGRA tissue expander (PMT Corp) was placed in the right thoracic cavity. It was inflated with 70 mL of saline via a subcutaneous port, while monitoring tracheal decompression under bronchoscopic guidance, resulting in significant relief of tracheal compression without any complications (Figure 1). Two days later, ASD closure was performed via median sternotomy. Adequate exposure was achieved by mobilizing the main pulmonary artery leftward, and the ASD was directly closed through a right atriotomy under cardiac arrest. Aortopexy was unnecessary, as the trachea remained expanded. The patient was extubated the next day and recovered uneventfully.

**Figure 1:**
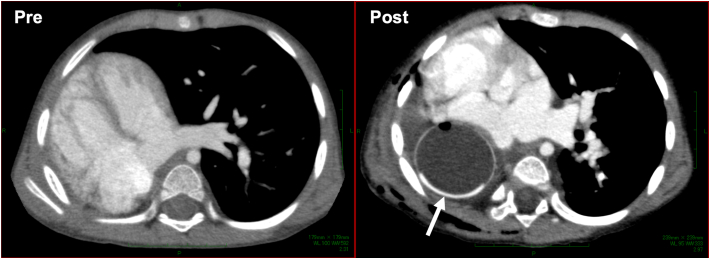
Computed tomography images before (left) and after (right) intrathoracic tissue expander insertion. Arrow indicates the tissue expander.

At 5 years old, routine echocardiography revealed a highly mobile right atrial mass and compression of the right atrium by the tissue expander (Figure 2). Due to embolism risk, urgent surgery was performed. Via median resternotomy and right atriotomy under cardiac arrest, a 10×15 mm mass attached near the superior vena cava orifice was removed (Video). Histology confirmed an organized thrombus. The patient was discharged on postoperative day 11 with aspirin.

**Figure 2:**
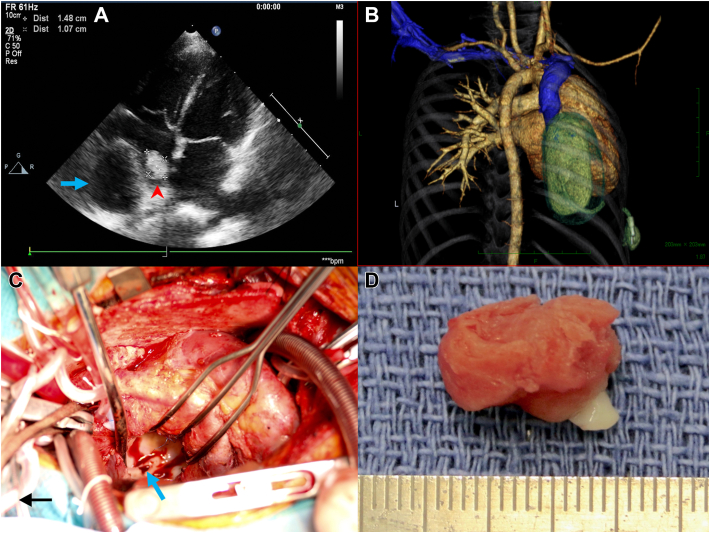
**(A)** Preoperative transthoracic echocardiogram demonstrating an intracardiac mass within the right atrium (red arrowhead) and the adjacent tisskue expander (blue arrow). (B) Three-dimensional reconstructed computed tomography image illustrating right atrial compression by the tissue expander (highlighted in green). (C) Intraoperative photograph showing the site of thrombus attachment within the right atrial wall (blue arrow). Black arrow indicates the head. (D) Gross specimen of the excised thrombus.

Due to persistent right atrial compression by the expander, it was partially deflated under bronchoscopic guidance 2 months after surgery. The trachea remained stable after deflation. As no respiratory symptoms developed, the expander was completely removed 15 months after thrombectomy (Figure 3). During an 8-year follow-up period, there was no recurrence of thrombus or respiratory complications.

**Figure 3:**
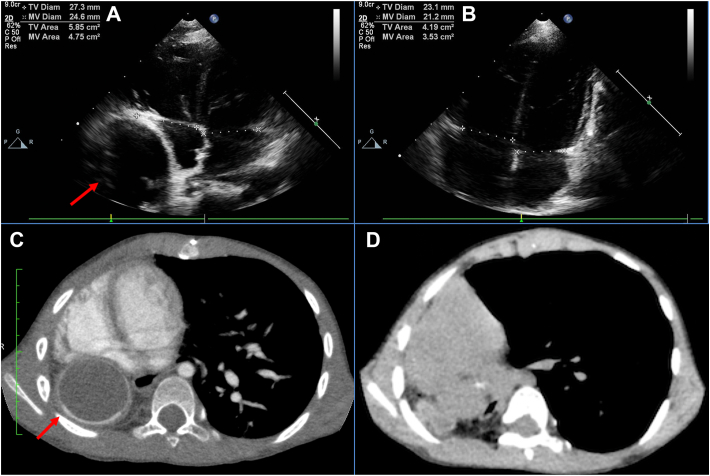
Transthoracic echocardiography (A, B) and computed tomography (C, D) before (left: A, C) and after (right: B, D) expander deflation. Arrows indicate the tissue expander.

## Comment

Pulmonary agenesis is a rare congenital anomaly often associated with cardiopulmonary malformations. Mediastinal shift toward the affected side can result in airway compression and altered cardiovascular dynamics, sometimes necessitating interventions to stabilize the trachea and restore hemodynamic balance.^4^ Although tissue expanders are used in thoracic surgery, their role in pediatric cardiac surgery is rarely reported. In this case, the expander served a dual purpose: improving respiratory stability and enabling adequate exposure of cardiovascular structures for ASD closure via median sternotomy.

Given the evolution of surgical approaches, particularly minimally invasive techniques such as right axillary thoracotomy, the need for intrathoracic expanders in similar cases may now be obviated. There have been reports of successful median sternotomy without the use of tissue expanders in patients with pulmonary agenesis; however, these cases often required modified exposure techniques due to limited access and distorted mediastinal anatomy.^2^^,^^5^^,^^6^ In contrast, our approach using a tissue expander allowed straightforward exposure of the great vessels and atrial structures under standard median sternotomy, facilitating a safe and effective surgical procedure. In patients with pulmonary agenesis, minimizing compression of the functional lung is also critical; the controlled mediastinal repositioning achieved with the tissue expander contributed to preserving contralateral pulmonary function.

However, 3 years after expander placement and ASD closure, the patient developed a right atrial thrombus caused by sustained external compression of the right atrium. The elimination of left-to-right shunting may have lowered right atrial pressure, making it more susceptible to extrinsic compression and flow disturbance, leading to thrombus formation. Cardiac compression has previously been noted as a perioperative risk during tissue expander insertion^7^; our case highlights that such compression can also have delayed hemodynamic consequences, including thrombus formation. This case underscores the risks associated with off-label use of devices like tissue expanders. While effective perioperatively, long-term effects on cardiac function may be unforeseen, necessitating vigilant and extended follow-up.

In conclusion, the use of a tissue expander in this pediatric case yielded important short-term benefits, including effective tracheal decompression and facilitation of cardiac surgery. However, the subsequent development of a right atrial thrombus highlights the need for sustained postoperative surveillance. Given the off-label nature of such interventions, their long-term safety must be carefully assessed in the context of evolving surgical techniques and patient-specific anatomical considerations.
